# Poor outcome is associated with less negative fluid balance in patients with aneurysmal subarachnoid hemorrhage treated with prophylactic vasopressor-induced hypertension

**DOI:** 10.1186/s13613-016-0128-6

**Published:** 2016-03-31

**Authors:** Yasser Sakr, Pedro Dünisch, Clesar Santos, Lena Matthes, Mohamed Zeidan, Konrad Reinhart, Rolf Kalff, Christian Ewald

**Affiliations:** Department of Anesthesiology and Intensive Care, Uniklinikum Jena, Erlanger Allee 101, 07743 Jena, Germany; Department of Neurosurgery, Uniklinikum Jena, Erlanger Allee 101, 07743 Jena, Germany; Department of Intensive Care, Federal University of Espírito Santo, R. Natalina D. Carneiro 874, Vitória, 29060490 Brazil; Department of Anaesthesiology and Intensive Care, Theodor Bilharz Institute, Cairo, Egypt; Department of Anesthesiology and Intensive Care, Friedrich-Schiller-University, Erlanger Allee 101, 07743 Jena, Germany

**Keywords:** Subarachnoid hemorrhage, Outcomes, Negative fluid balance

## Abstract

**Background:**

Aneurysmal subarachnoid hemorrhage (SAH) is a serious condition associated with high mortality rates and long-term disability. We investigated the impact of fluid balance on neurologic outcome after adjustment for possible confounders related to intensive care therapy and extra-cerebral organ failure during the early phase after SAH.

**Methods:**

In this retrospective study, we analyzed data from all 142 adult patients admitted to our university hospital surgical intensive care unit (ICU) with SAH between March 2004 and November 2010.

**Results:**

The mean patient age was 54 ± 14 years, 62.7 % were female, and the median Hunt and Hess score was 3. The proportions of patients with poor outcome (Glasgow Outcome Score ≤3) were 58.4, 54.2, and 52.1 % at 3, 6, and 12 months, respectively, after the SAH. The ICU and hospital mortality rates were both 12.7 %, and the median lengths of stay in the ICU and the hospital were 16 (IQ 7–25) and 26 (IQ 18–34) days, respectively. In multivariable analysis, older age and greater cumulative fluid balance within the first 7 days in the ICU were independently associated with a greater risk of poor outcome.

**Conclusion:**

In this cohort of patients, older age and greater cumulative fluid balance were independently associated with a greater risk of poor outcome up to 1 year after the initial insult. Our data suggest that mild hypovolemia may be beneficial in the management of these patients.

**Electronic supplementary material:**

The online version of this article (doi:10.1186/s13613-016-0128-6) contains supplementary material, which is available to authorized users.

## Background

Aneurysmal subarachnoid hemorrhage (SAH) is a serious condition, not only because of the relatively high associated mortality rates, but also because it is common in young adult patients, leading to long-term disability and loss of productivity [1]. The course of the disease can be prolonged, with considerable secondary brain injury as a result of delayed cerebral ischemia [2].

The ultimate goal of therapy in patients with acute SAH is to control bleeding, provide supportive care in the acute phase, maintain adequate cerebral perfusion, and prevent secondary cerebral insults, especially vasospasm and cerebral infarction [1, 3]. The “triple-H” therapy, including hypervolemia, hypertension, and hemodilution, has been proposed as an effective approach in the management of patients with SAH. However, several randomized controlled studies have demonstrated that triple-H therapy does not improve cerebral blood flow, nor does it have a positive impact on the neurologic outcome of patients with SAH [4, 5]; indeed, this approach may be harmful and increase therapeutic costs [5–7]. Hypervolemic therapy may, in particular, be associated with high rates of complications, including pulmonary edema, dilutional hyponatremia, coagulopathy, and aneurysm rebleeding [8]. The possible confounding effects of other intensive care therapies, especially vasopressor and fluid regimens, as well as the impact of extra-cerebral organ failure during the early phase after SAH, have not been systematically considered in the previous literature.

The aim of our study was, therefore, to investigate the impact of fluid balance on neurologic outcome after adjustment for possible confounders related to intensive care therapy and extra-cerebral organ failure during the early phase after SAH. We hypothesized that mild hypovolemia would be associated with good neurologic outcome in these patients.

## Methods

We included all adult (>18 years old) patients admitted to our 50-bed surgical intensive care unit (ICU) with SAH from March 2004 until November 2010. Patients were identified retrospectively using codes from the International Classification of Diseases-10 (ICD-10).

### Ethics, consent, and permissions

The study was approved by the institutional review board of Friedrich Schiller University Hospital, Jena, Germany (Bachstrasse 18, 07743 Jena). Informed consent was waived due to the retrospective, anonymous nature of the analysis.

### Data collection

Two investigators (LM and PD) reviewed the medical charts and computed tomography (CT) images of patients with SAH during the study period. The Hunt and Hess score [9] (Additional file 1: Table S1), recorded prospectively by the referring physician at the onset of symptoms, was noted. The World Federation of Neurological Surgeons clinical grading scale (WFNS) [10] was calculated retrospectively using the initial Glasgow Coma Scale (GCS) score [11]. The extent of the SAH was graded according to the Fisher scale [12] (Additional file 1: Table S1), which assesses the amount of bleeding seen on the initial head CT scan. Complications related to SAH were also recorded and included the occurrence of vasospasm, hydrocephalus, and cerebral infarction (confirmed by CT imaging).

Physiologic, hemodynamic, and therapeutic data from the ICU stay were collected and automatically recorded by a patient data management system (Copra System GmbH, Sasbachwalden, Germany). Data recorded prospectively on admission included age, sex, primary and secondary admission diagnoses, and surgical procedures. The Simplified Acute Physiology Score (SAPS) II [13] was calculated on admission, and the Sequential Organ Failure Assessment (SOFA) score [14] was calculated daily by the physician in charge of the patient using a special sheet. In sedated patients, the GCS measured prior to initiation of sedation was considered.

### Management of patients with SAH

An initial cerebral CT scan was performed routinely in all patients on arrival to the emergency room, after hemodynamic stabilization. In patients with CT evidence of increased intracranial pressure (ICP), massive hemorrhage (Hunt and Hess grade 4), brain edema, and no possibility for appropriate neurologic assessment because of ongoing analgosedation or severe impairment of the conscious level (GCS < 8), extraventricular drains were surgically inserted. In some patients with massive brain edema or hemorrhage, craniectomy was performed. Cerebral angiography was performed in all patients within 12 h of admission to the hospital.

On admission to the ICU, blood samples were routinely drawn to obtain a complete blood picture, serum creatinine, blood urea, C-reactive protein, bilirubin levels, activated prothrombin time (aPTT), and serum electrolytes (sodium and potassium). These parameters were measured at least once daily thereafter (at 6:00 a.m.) during the ICU stay. Fluid intake (enteral and parenteral) and output (urinary output, drains, and gastrointestinal losses) were measured and recorded hourly in the electronic charts. Insensible water loss was estimated hourly by the nurse in charge of the patient according to body mass index, body temperature, and the presence of mechanical ventilation [15].

According to our standard operative procedures (SOPs), patients with SAH were kept with a head-elevated position of 30°–45° with the head aligned to the body axis to avoid venous congestion. Pressure transducers were placed at the level of the ears. Central venous pressure (CVP) was measured and recorded every 4 h in the neutral position. In mechanically ventilated patients, the minute volume was adjusted to keep PCO_2_ within the normocapnic range (4.7–6.0 kPa). Patients with external ventricular drains had continuous monitoring of ICP and cerebral perfusion pressure (CPP), which was displayed as the difference between mean arterial and ICP. Vasospasm was assessed using transcranial Doppler (TCD) on a daily basis and, if present, graded into mild (≥120 <160 cm/s), moderate (≥160 <200 cm/s), or severe (≥200 cm/s) according to the maximal flow in any vessel. A cerebral CT scan was performed routinely after each intervention if there was clinical suspicion of intracranial complications, or every 3 days during the first 2 weeks after hospital admission to guide therapy.

The mean arterial blood pressure target was 70–80 mmHg for the first 3 days after SAH or to maintain a CPP > 65 mmHg in patients with external ventricular drains. The target was set at a higher level after the third day to reach 80–90 mmHg in patients without evidence of vasospasm and between 90 and 100 mmHg in those with vasospasm for up to 2 weeks or until the vasospasm had resolved. These targets were achieved by the infusion of incremental doses of norepinephrine. In patients requiring high doses of norepinephrine (>0.4 µg/kg/min), additional boli of crystalloids were considered at the discretion of the attending physician.

The target for fluid administration was to keep the daily fluid balance at zero or slightly negative (500–1000 ml/day). This was achieved using infusion of balanced full-electrolyte infusions (E153™; Serumwerk Bernburg AG, Bernburg, Germany, or Jonosteril™; Fresenius Kabi GmbH, Bad Homburg, Germany) at a basic rate of 1000–1500 ml/day. Colloid solutions were additionally used in some patients when clinically indicated in the form of 6 % hydroxyethyl starch (130/0.4, Voluven™; Fresenius Kabi, Bad Homburg, Germany). Since June 30, 2005, 4 % gelatin solutions (Gelafusal™; Serumwerk Bernburg AG, Bernburg, Germany) have replaced hydroxyethyl starch as the colloid of choice in our institution. Synthetic colloids have been completely banned in our ICU since 2008.

### Outcome assessment

Sepsis was defined as the presence of infection, documented or supposed, plus systemic manifestations of this infection, and severe sepsis was defined as sepsis with associated organ failure [16]. All patients who survived the initial hospital stay were routinely given follow-up appointments at the neurosurgery polyclinic at 3, 6, and 12 months after SAH at which the neurologic status was examined and documented. Nine patients were lost to follow-up, but we were able to contact their next-of-kin by telephone to obtain the necessary information about neurologic outcome. The Glasgow Outcome Score (GOS) [17] was calculated retrospectively according to the available data: grade 1, death; grade 2, persistent vegetative state; grade 3, severe disability; grade 4, moderate disability, somewhat disabled but still independent for daily life; and grade 5, good recovery, although minor neurologic or psychological deficits could be present. Patients were stratified as having a poor (GOS ≤ 3) or good (GOS 4–5) outcome at 3, 6, and 12 months.

### Statistical analysis

Data were analyzed using SPSS 16.0 for windows (SPSS Inc, Chicago, IL, USA). Nonparametric tests of comparison were used for variables evaluated as not being normally distributed. Difference testing between groups was performed using Student’s *t* test, Mann–Whitney *U* test, Chi-square test, or Fisher’s exact test as appropriate. Analysis of variance (ANOVA) was used to assess differences between groups over time with a post hoc Mann–Whitney *U* test to assess the differences at each time point.

To define the possible factors associated with poor outcome in SAH, we performed multivariable logistic regression analyses, with poor outcome (GOS ≤ 3) at 3, 6, and 12 months after the SAH, as the dependent variable. The variables considered for this analysis were age, sex, SAPS II on admission to the ICU, the maximum degree of extracranial organ dysfunction/failure as assessed by the maximum SOFA subscores during the ICU stay, duration of mechanical ventilation, the initial WFNS and Hunt and Hess score, the Fisher score from the first CT scan following the onset of SAH, localization of the ruptured aneurysm, the type of primary intervention, the mean hemoglobin level and the lowest mean arterial blood pressure during the first week in the ICU, the occurrence of infection or severe sepsis during the ICU stay, the cumulative fluid balance and the maximum dose of norepinephrine within the first week in the ICU, the use of colloids during the ICU stay, the occurrence of complications (vasospasm, hydrocephalus, and cerebral infarction) during the ICU stay, and the maximum sodium concentration during the ICU stay. To reduce the number of covariates in the multivariable models, we adopted a forward, stepwise approach with entry and removal based on a univariate *p* value of 0.2. Collinearity between variables was excluded before modeling (*R*^2^ > 0.6), and none of the covariates was colinear. A Hosmer and Lemeshow goodness-of-fit test was performed, and odds ratios (OR) with 95 % confidence intervals (CI) were computed.

All statistics were two tailed. A *p* value <0.05 was considered to be significant. Continuous variables are presented as mean ± standard deviation or median [25–75 % interquartile range (IQ)] and categorical variables as number and percentage, unless otherwise indicated.

## Results

### Characteristics of the study cohort

During the study period, 142 patients were admitted to our ICU after aneurysmal SAH [mean age 54 (SD 14) years, 62.7 % female]. The characteristics of the study group on admission to the ICU are given in Table 1. The median Hunt and Hess score was 3 (IQ 2–4), and the median WFNS was 3 (2–4). Aneurysms of the anterior circulation were responsible for 87.4 % of the cases (Additional file 1: Table S2). Surgical clipping was performed in 102 patients (71.8 %) and external ventricular drainage in 131 patients (92.3 %) (Additional file 1: Table S2).

**Table 1:** Characteristics of the study cohort on admission to the ICU

	n = 142
Age, years, mean ± SD	54 ± 14
Sex, female, *n* (%)	89 (62.7)
Source of admission, *n* (%)	
Emergency room	49 (34.5)
Transferred from another hospital	93 (65.5)
Hunt and Hess score, *n* (%)	
1	10 (7.0)
2	36 (25.4)
3	39 (27.5)
4	25 (17.6)
5	32 (22.5)
WFNS-classification, *n* (%)	
1	35 (24.6)
2	33 (23.2)
3	13 (9.2)
4	29 (20.4)
5	32 (22.5)
GCS, median (IQ)	13 (7–14)
Fisher’s score, *n* (%)	
1	1 (0.7)
2	17 (12.0)
3	32 (22.5)
4	92 (64.8)
Severity scores, mean ± SD	
SAPS II	37.9 ± 14.5
SOFA score	5.9 ± 2.7

*GCS* Glasgow Coma Scale, *IQ* interquartile range, *SAPS II* Simplified Acute Physiology Score II, *SD* standard deviation, *SOFA* sequential organ failure assessment, *WFNS* World Federation of Neurological Surgeons

### Morbidity and mortality

Vasospasm was the most commonly reported complication and occurred in 65.5 % of patients (*n* = 93). Moderate and severe vasospasm were present in 43.8 and 32.6 % of patients, respectively, after a median of 5 days (IQ 3–7) and lasted for a median of 9 days (IQ 6–17). Hydrocephalus occurred in 83 (58.5 %) and cerebral infarction in 81 (57.0 %) patients (Table 2). The ICU and hospital mortality rates were both 12.7 %, and the median lengths of stay in the ICU and the hospital were 16 (IQ 7–25) and 26 (IQ 18–34) days, respectively. At 3, 6, and 12 months after onset of the aneurysmal SAH, 58.4, 54.2, and 52.1 % of patients, respectively, had a poor outcome (GOS ≤ 3).

**Table 2:** Morbidity and mortality

Cerebral infarction	
*n* (%)	81* (57.0)
Territory, *n* (%)	
Anterior cerebral artery	50 (61.7)
Middle cerebral artery	67 (82.7)
Posterior cerebral artery	10 (12.3)
Vasospasm-related infarction	54 (42.5)
Hydrocephalus, *n* (%)	83 (58.5)
Vasospasm	
*n* (%)	93^†^ (65.5)
Localization, *n* (%)	
Anterior cerebral artery	47 (50.5)
Middle cerebral artery	87 (93.5)
Posterior cerebral artery	1 (1.1)
Basilar artery	1 (1.1)
Severity of vasospasm^‡^, *n* (%)	
Mild (≥ 120 < 160 cm/s)	21 (23.6)
Moderate (≥ 160 < 200 cm/s)	39 (43.8)
Severe (≥ 200 cm/s)	29 (32.6)
Mortality rate, *n* (%)	
ICU mortality	18 (12.7)
Hospital mortality	18 (12.7)
ICU stay (days) median (IQ)	16 (7–25)
Hospital stay (days) median (IQ)	26 (18–34)
Glasgow Outcome Score ≤3	
After 3 months	83 (58.4)
After 6 months	77 (54.2)
After 12 months	74 (52.1)

*ICU* intensive care unit, *IQ* interquartile range, *TCD* transcranial Doppler

* 41 patients with one infarcted area, 34 patients with two infarcted areas, and 6 patients with 3 infarcted areas

^†^50 patients with one area of vasospasm and 43 patients with two areas of vasospasm; 76 cases were detected with TCD; 13 detected with TCD and angiography; and 4 detected only through angiograph

### The impact of SAH-related factors on outcome

Patients who had a poor outcome (GOS ≤ 3) at 3, 6, and 12 months were older, had higher Hunt and Hess, WFNS and Fisher scores, and more commonly required external ventricular drainage, craniectomy, and ventriculoperitoneal shunts compared with those who had good outcomes (GOS 4–5) (Additional file 1: Table S3). The frequencies of hydrocephalus and cerebral infarction were higher in patients with poor than in those with good outcome at 3, 6, and 12 months (Additional file 1: Table S4). Although the occurrence of vasospasm was similar, the severity of vasospasm was worse in patients with poor outcome compared with those with good outcome throughout the follow-up period (Additional file 1: Table S4).

### The impact of severity of illness, organ function, and ICU interventions on outcome

**Fig. 1:**
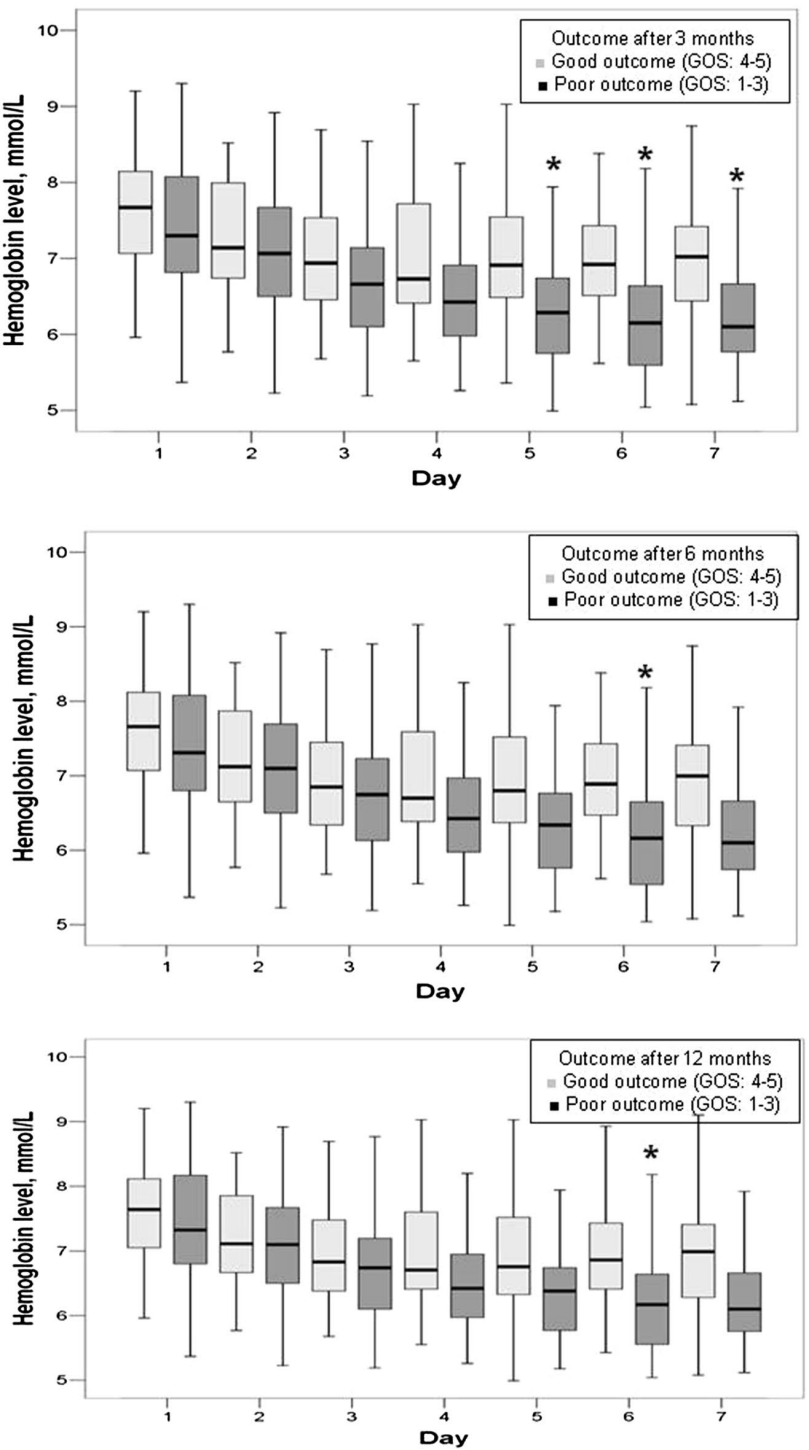
*Box blots* representing the time course of hemoglobin levels in mmol/l over the first week in the ICU in patients with poor (Glasgow Outcome Score; GOS ≤ 3, *dark boxes*) versus good (GOS > 3, *light boxes*) outcome at 3, 6, and 12 months after the onset of SAH. ANOVA: 3 months: *p* = 0.085, 6 months: *p* = 0.125, 12 months: *p* = 0.114. *p < 0.05 compared with patients with good outcome

SAPS II and SOFA scores and the maximum sodium concentration within 24 h of admission to the ICU were higher in patients with poor than in those with good outcomes after 3, 6, and 12 months (Table 3). Hemoglobin levels were similar irrespective of outcome over the first 4 days in the ICU and were higher thereafter in patients who had a good outcome compared with those with poor outcomes (Fig. 1). Patients who had a poor outcome after 3, 6, and 12 months were more likely to need mechanical ventilation and vasopressor support to maintain the target blood pressure during the ICU stay than patients with good outcomes (Additional file 1: Table S5), but the doses of vasopressors (norepinephrine) were similar in the two groups. Infections, predominantly those of respiratory origin, and severe sepsis occurred more commonly, and the degree of organ dysfunction/failure was greater in patients who had poor compared with those with good outcomes throughout the follow-up period (Additional file 1: Table S5). Patients who had a poor outcome had a greater incidence of respiratory and cardiovascular failure during the ICU stay compared with patients who had good outcomes (Additional file 1: Table S5).

**Table 3:** Severity of illness, use of vasopressors, need for mechanical ventilation, and physiologic parameters on admission to the ICU according to the GOS

	GOS after 3 months		GOS after 6 months		GOS after 12 months	
	GOS 1–3	GOS 4–5	GOS 1–3	GOS 4–5	GOS 1–3	GOS 4–5
SAPS II, mean ± SD	42.2 ± 13.2	31.6 ± 14.1^‡^	42.5 ± 13.3	32.3 ± 14.0^‡^	42.6 ± 13.2	32.6 ± 14.1^‡^
SOFA score, total, mean ± SD	6.7 ± 2.3	4.8 ± 2.7^‡^	6.8 ± 2.3	4.9 ± 2.8^‡^	6.7 ± 2.2	5.0 ± 2.9^‡^
SOFA score, without GCS, mean ± SD	4.6 ± 2.4	2.9 ± 2.2^‡^	4.6 ± 2.4	3.0 ± 2.3^‡^	4.6 ± 2.5	3.1 ± 2.3^†^
Physiologic parameters, median (IQ)						
Creatinine (µmol/l)	68 (61–81)	68 (70–79)	69 (61–83)	67 (60–77)	70 (61–84)	67 (61–77)
Bilirubin (µmol/l)	9 (7–14)	10 (8–14)	9 (7–14)	10 (7–14)	9 (7–14)	10 (8–15)
Platelets (1000/µl)	209 (183–266)	218 (197–248)	205 (180–265)	219 (198–251)	204 (178–267)	220 (198–248)
aPTT (s)	30 (28–33)	29 (27–35)	30 (28–33)	30 (27–35)	30 (27–33)	30 (27–36)
Leukocytes (Tsd/µl)	13.5 (10.5–17.0)	11.8 (9.4–14.8)	13.5 (10.7–17.1)	11.8 (9.2–15.2)	13.5 (10.7–17.0)	11.9 (9.2–15.3)
C-reactive protein (mg/l)	7.8 (3.4–16.0)	6.3 (3.0–15.0)	8.1 (3.4–16.1)	6.3 (3.0–14.9)	7.5 (3.4–15.5)	6.6 (3.0–15.1)
Hemoglobin (mmol/l)	7.8 (7.2–8.6)	8.2 (7.7–8.4)	7.8 (7.2–8.6)	8.2 (7.6–8.4)	7.8 (7.2–8.6)	8.2 (7.6–8.4)
Hematocrit	0.36 (0.33–0.40)	0.37 (0.34–0.39)	0.36 (0.33–0.40)	0.37 (0.34–0.39)	0.36 (0.33–0.40)	0.37 (0.34–0.39)
Sodium (min) (mmol/l)	139 (137–142)	138 (137–139)	139 (137–142)	138 (137–139)	139 (137–142)	138 (137–140)
Sodium (max) (mmol/l)	145 (142–148)	142 (141–144)^‡^	145 (143–148)	142 (141–144)^‡^	145 (143–148)	142 (141–145)^‡^

*aPTT* activated partial thromboplastin time

^†^ *p* < 0.01; ^‡^ *p* < 0.001

**Fig. 2:**
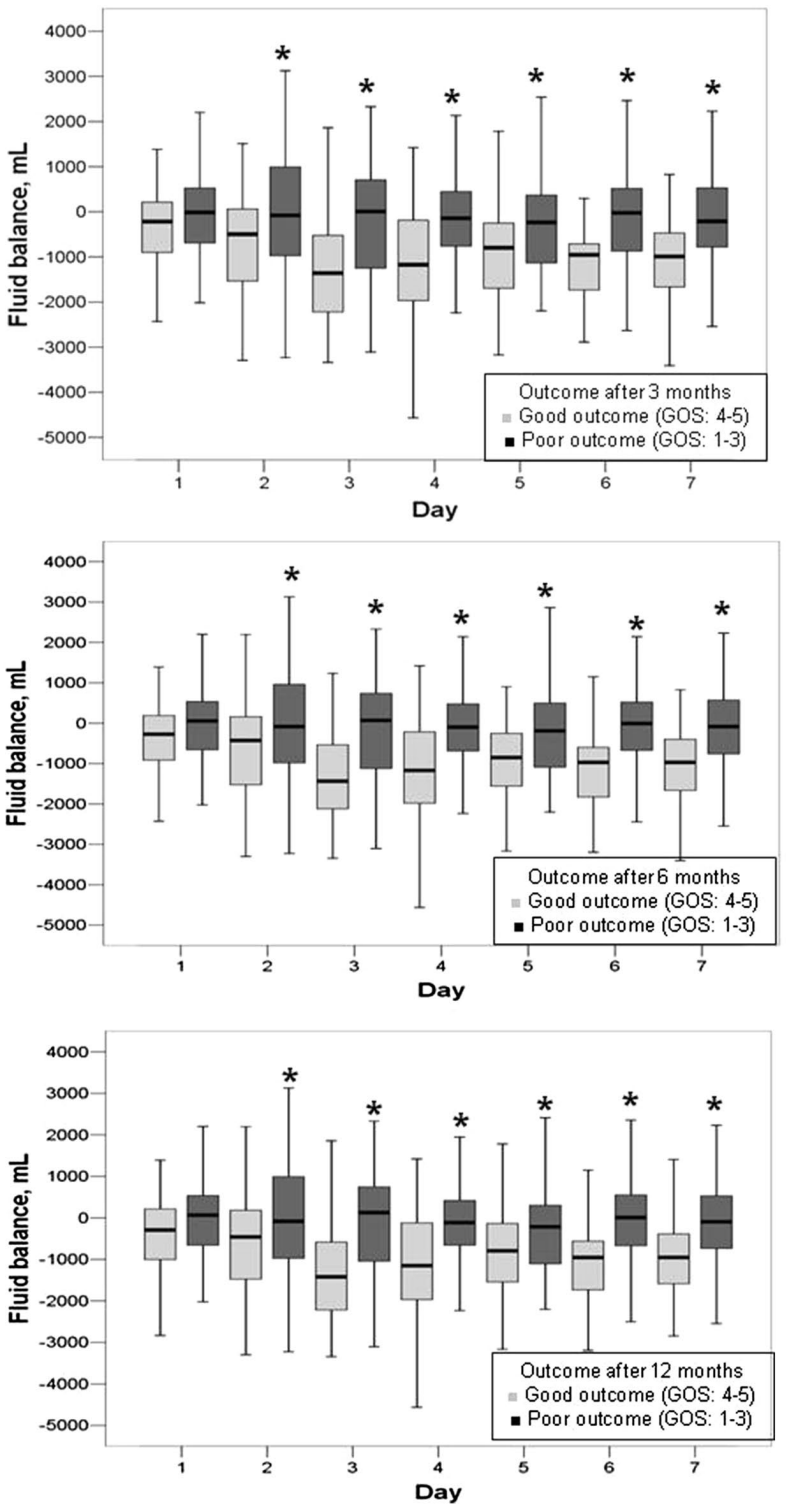
*Box blots* representing fluid balance in ml over the first week in the ICU in patients with poor (Glasgow Outcome Score; GOS ≤ 3, *dark boxes*) versus good (GOS > 3, *light boxes*) outcome at 3, 6, and 12 months after the onset of SAH. ANOVA: *p* < 0.001 at 3, 6, and 12 months. *p < 0.05 compared with patients with good outcome

**Fig. 3:**
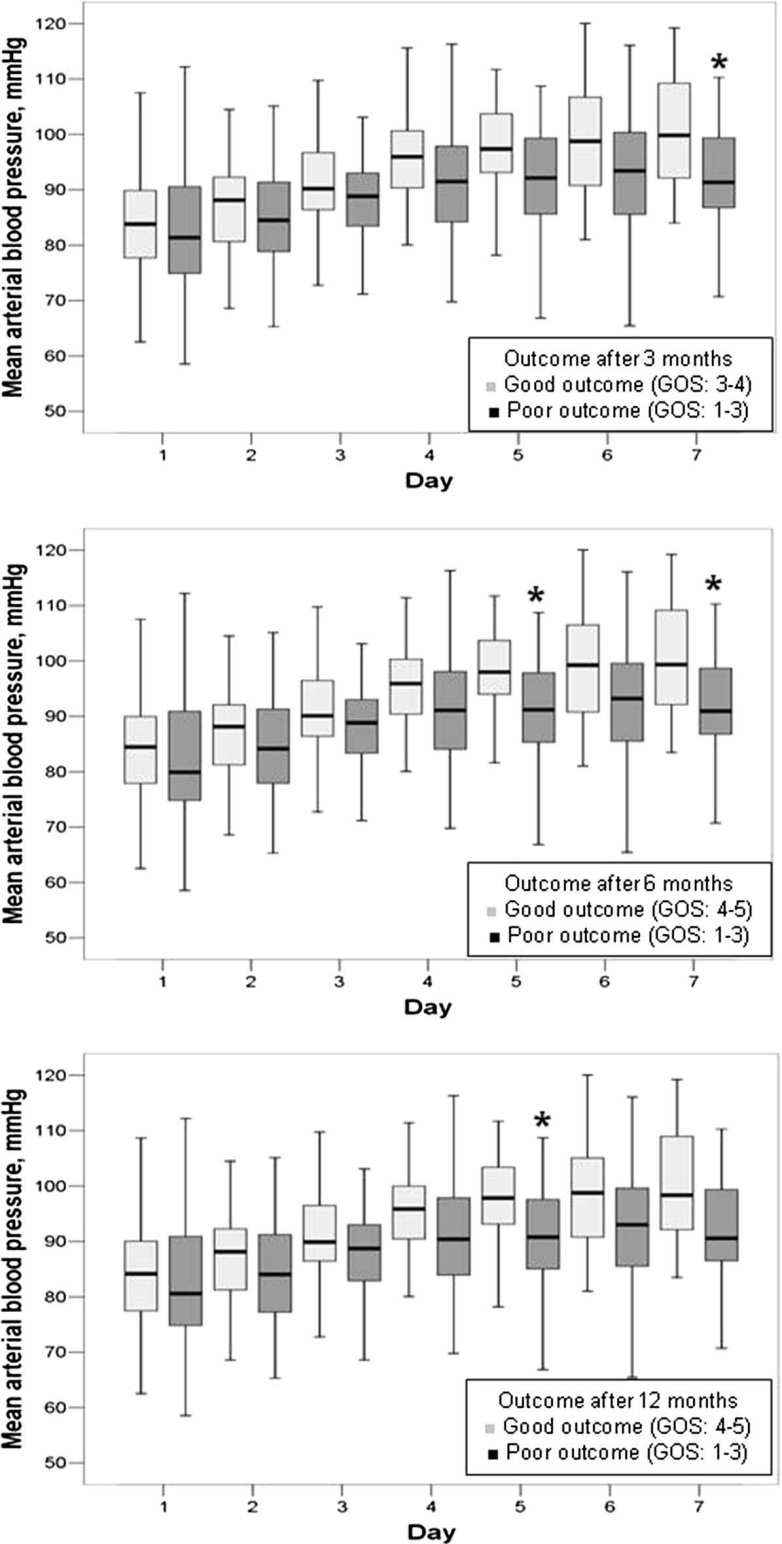
*Box blots* representing the mean arterial pressure in mmHg over the first week in the ICU in patients with poor (Glasgow Outcome Score; GOS ≤ 3, *dark boxes*) versus good (GOS > 3, *light boxes*) outcome at 3, 6, and 12 months after the onset of SAH. ANOVA: 3 months: *p* = 0.258, 6 months: *p* = 0.163, 12 months: *p* = 0.125. *p < 0.05 compared with patients with good outcome

Fluid balance within 24 h of admission to the ICU was similar in patients who had poor and those who had good outcomes at 3, 6, and 12 months; however, patients who had good outcomes achieved more negative fluid balances during the ICU stay up to the 7th day (Fig. 2; Table 4), despite markedly lower rates of diuretic therapy. This was mainly achieved by higher urinary outputs in patients who had good compared with those who had poor outcomes (Additional file 1: Figure S1). CVP values were similar within 24 h of admission to the ICU but were consistently lower in patients who had good compared with those who had poor outcomes up to 7 days in the ICU (Table 4 and Additional file 1: Figure S2). The mean arterial blood pressure was similar during the first 4 days in the ICU but was higher thereafter, up to 7 days in the ICU, in patients who had good compared with those who had poor outcomes (Fig. 3).

**Table 4:** Infused volumes and fluid balance according to the Glasgow Outcome Score (GOS)

	GOS after 3 months		GOS after 6 months		GOS after 12 months	
	GOS 1–3	GOS 4–5	GOS 1–3	GOS 4–5	GOS 1–3	GOS 4–5
Fluid balance, median (IQ)						
Cumulative (day, ml)	−223 ((−600)−22)	−806 ((−1228)–(−324))^‡^	−215 ((−585)−39)	−806 ((−1216)–(−332))^‡^	− 214 ((−582)−54)	−770 ((−1202)–(−328))^‡^
Within the first 7 days (l)	−0.7 ((−3.0)−1.2)	−5.6 ((−8.5)–(−1.7))^‡^	−0.6 ((−2.8)−1.3)	−5.8 ((−8.3)–(−1.8))^‡^	−0.6 ((−2.8)−1.2)	−5.7 ((−8.1)–(−1.6))^‡^
Cumulative in the ICU (l)	−5.9 ((−13.0)−0.5)	−8.7 ((−14.0)–(−2.2))	−5.3 ((−12.6)−0.5)	−8.7 ((−14.3)–(−2.4))*	−5.2 ((−12.4)−0.5)	−8.7 ((−14.4)–(−2.3))*
Colloids						
*n* (%)	54 (65.1)	32 (54.2)	50 (64.9)	36 (55.4)	47 (63.5)	39 (57.4)
Total (l), median (IQ)	4.0 (1.5–8.1)	3.8 (2.0–6.7)	4.0 (1.9–8.1)	3.5 (1.6–6.7)	4.0 (1.5–8.0)	4.0 (2.0–7.5)
Total (kg BW, ml), median (IQ)	51 (21–99)	44 (25–107)	51 (22–99)	44 (22–107)	42 (21–94)	53 (25–116)
Diuretics, *n* (%)						
Any	55 (66.3)	19 (32.2)^‡^	53 (66.8)	21 (32.3)^‡^	50 (67.6)	24 (35.3)^‡^
Furosemide/bolus (10–20 mg)	47 (65.6)	16 (27.1)^‡^	45 (58.4)	18 (27.7)^‡^	42 (56.8)	21 (30.9)^‡^
Furosemide/perfusion (2–5 mg/h)	16 (19.3)	3 (5.1)*	15 (19.5)	4 (6.2)*	13 (17.6)	6 (8.8)
Others	21 (25.3)	6 (10.2)*	21 (27.5)	6 (9.2)^†^	21 (28.4)	6 (8.8)^†^
CVP (mmHg), median (IQ)						
Within 24 h	6 (4–7.9)	6 (5–8.4)	6 (4–8.3)	6 (5–8.1)	6 (4–8.3)	6 (5–8.1)
Within 7 days	7 (5.5–7.8)	5 (4.3–6.3)^‡^	7 (5.5–7.9)	5 (4.3–6.4)^‡^	7 (5.5–8)	5 (4.5–6.5)^‡^

*CVP* central venous pressure

* *p* 0.05–0.01; ^†^ *p* < 0.001; ^‡^ *p* < 0.001

### Multivariable adjustment

In multivariable analysis, older age and greater cumulative fluid balance within the first 7 days in the ICU were independently associated with a higher risk of poor outcome throughout the follow-up period (Table 5). Higher Fisher scores on hospital admission and the duration of mechanical ventilation during the ICU stay were independently associated with a higher risk of poor outcome at 3 and 6 months. Higher WFNS on hospital admission, higher sodium levels in the ICU, and the occurrence of infection during the ICU stay were additionally found to be associated with an increased risk of poor outcome at 1 year after the onset of SAH.

**Table 5:** Summary of multivariable analysis^a^ with poor outcome (GOS ≤ 3) as the dependent variable

	After 3 months^†^		After 6 months^‡^		After 12 months^¶^	
	OR (95 % CI)	*p* value	OR (95 % CI)	*p* value	OR (95 % CI)	*p* value
Age (per year)	1.13 (1.06–1.20)	<0.001	1.13 (1.06–1.21)	<0.001	1.08 (1.03–1.14)	0.003
Fluid balance within 7 days (per l)	1.24 (1.08–1.42)	0.002	1.21 (1.06–1.39)	0.006	1.19 (1.04–1.36)	0.011
Fisher’s score (per point)	7.41 (2.43–22.57)	<0.001	5.33 (1.59–17.8)	0.007	2.40 (0.90–6.38)	0.079
Mechanical ventilation (per day)	1.10 (1.04–1.16)	<0.001	1.10 (1.04–1.18)	0.002	–	–
WFNS score (per point)	–	–	1.59 (0.97–2.61)	0.064	1.84 (1.15–2.93)	0.011
Maximum sodium levels in the ICU (per mmol/l)	–	–	–	–	1.2 (1.03–1.39)	0.021
Infection during the ICU stay	–	–	–	–	7.14 (2.18–23.39)	0.001

*CI* confidence interval, *OR* odds ratio

^†^Hosmer and Lemeshow Chi-square = 5.342 (*p* = 0.721); pseudo-*R* ^2^ 0.699

^‡^Hosmer and Lemeshow Chi-square = 5.11 (*p* = 0.746); pseudo-*R* ^2^ 0.738

^¶^Hosmer and Lemeshow Chi-square = 6.586 (*p* = 0.582); pseudo-*R* ^2^ 0.68

^a^Forward stepwise logistic regression analysis with poor outcome (GOS ≤ 3) as the dependent variable. Only variables that were retained at the final step are displayed

## Discussion

The main findings of our study were that in patients with aneurysmal SAH: (1) patients who had good outcomes achieved more negative fluid balances after the day of admission up to the 7th day during the ICU stay than those who had poor outcomes; (2) the mean arterial blood pressure was similar during the first 4 days in the ICU but was higher thereafter in patients with good compared to those with poor outcomes for up to 7 days in the ICU; and (3) greater cumulative fluid balance within the first 7 days in the ICU was independently associated with a higher risk of poor outcome throughout the follow-up period.

Although the incidence of vasospasm was similar, the severity of vasospasm was higher in patients with poor outcomes compared with those with good outcomes throughout the follow-up period. This type of association has been reported consistently in previous studies [18–23]. Vasospasm is a major determinant of brain ischemia after SAH [24] and may be responsible for delayed neurologic deterioration and severe disability [22, 23]. As expected, the higher rates of vasospasm in our study in patients with poor outcome were associated with higher incidences of cerebral infarction and hydrocephalus as well as the need for insertion of external ventricular drains and decompression craniectomy compared with patients with good outcomes [25, 26]. The relatively high prevalence of vasospasm and cerebral infarctions in our study can be explained by the severity of illness of our patients, as evident from the high Hunt and Hess score and WFNS. Our institution is the largest tertiary care referral center in the state of Thuringia, Germany, in which in-house neurosurgical and interventional radiologists are available 24 h/day. We therefore receive the most severe cases of SAH.

An interesting finding of our study was that greater cumulative fluid balance within the first 7 days in the ICU was independently associated with a higher risk of poor outcome, suggesting that mild hypovolemia may improve neurologic outcome in these patients. Indeed, fluid balance is not a surrogate for assessment of blood volume [2, 27] and CVP does not precisely reflect intravascular volume status [2, 28]. However, an expert panel has acknowledged that close monitoring of fluid input and output should be the primary assessment tool in these patients, in addition to physical findings and clinical data [2]. Although these recommendations also implied that euvolemia should be targeted in patients with SAH [2], current evidence does not refute the possible beneficial effects of mild hypovolemia on outcome in these patients. Maintaining normovolemia may necessitate the use of relatively large fluid volumes, which may lead to cerebral edema and negatively influence neurologic recovery. Although hypovolemia has been described as being associated with higher rates of cerebral infarction [29, 30], these observations were not systematically adjusted for possible confounding factors and did not take into account the degree and timing of hypovolemia. In a recent retrospective cohort, Martini et al. [21] found that positive fluid balance was associated with increased rates of vasospasm and also with increased ICU lengths of stay after aneurysmal SAH. Similar results have been reported in a more recent propensity score-matched analysis [31]. Unfortunately, these studies did not analyze the possible impact of negative fluid balance on outcome [21, 31].

The hemodynamic pattern following SAH is quite complex. Sympathetic hyperactivity may evoke a hyperdynamic and hypovolemic state [32]. Conversely, in a large multicenter cohort of 204 patients, hemodynamic measurements early after SAH revealed a heart failure-like afterload mismatch with rather elevated global end-diastolic volume index (GEDVI), signifying increased preload [33]. Nonetheless, decreased GEDVI and cardiac index, in addition to increased systemic vascular resistance, were independently associated with the risk of delayed cerebral ischemia [33]. Goal-directed therapy using transpulmonary thermodilution monitoring has been reported to be effective in reducing the frequency of vasospasm and cardiopulmonary complications compared with standard therapy, but it did not influence functional outcomes at 3 months [34]. The absence of a positive effect on outcome does not support routine aggressive volume administration and inotropic therapy to maintain euvolemia and high cardiac index. We may assume that mild permissive hypovolemia would be well tolerated and associated with good neurologic outcome early after SAH. This may be supported by vasopressor-induced hypertension as used in our institution during the study period. This approach has been reported to be safe, even in patients with coexisting unruptured, unprotected intracranial aneurysms [35]. Further randomized studies are needed to confirm this assumption.

In our study, hemoglobin levels were not found to have an impact on outcome after adjustment for possible confounders. Our data do not, therefore, support hemodilution as a therapeutic approach for aneurysmal SAH. As higher arterial blood pressure was the main target of our therapy and levels were maintained within the hypertensive range in all our patients, it is not possible to elaborate on the efficacy of using hypertensive therapy in these patients.

Our analysis has some limitations. First, a cause–effect relationship cannot be established with certainty because of the retrospective nature of the analysis and the inherent limitations of the multivariable analysis. However, our data should be considered to be hypothesis generating. Second, management of SAH may vary among institutions and geographic regions, which hinders the generalizability of our results. We also reported on patients with a relatively severe form of SAH admitted to a single center, and our findings may, therefore, not be applicable to patients with milder forms of the disease. However, the multivariable analyses account for the confounding effects of therapeutic interventions and the heterogeneity of case-mix. Moreover, the single-center nature of the study may be an advantage as it limits the impact of variations in practice that can be a confounding factor in multicenter studies. Third, the multivariable analysis considers only variables included in the statistical model, and the influence of unmeasured variables cannot be accounted for. Third, numerous comparisons have been reported in our study potentially creating type II errors. However, this is not expected to have influenced the results of the multivariable analysis, which is not subject to such errors. Finally, we were not able to assess symptomatic vasospasm due to the relatively large number of mechanically ventilated patients in our study. Nonetheless, current recommendations on the management of patients with SAH suggest reporting only radiographic evidence of cerebral infarction and functional outcome as the primary outcome measures in these patients as the term “symptomatic vasospasm” implies the use of some subjective criteria, which may be difficult to assess in clinical practice [2].

## Conclusion

In this cohort of patients with aneurysmal SAH, older age and greater cumulative fluid balance within the first 7 days in the ICU were independently associated with a greater risk of poor outcome for up to 1 year after the initial insult. Our data suggest that mild hypovolemia may be beneficial in the treatment of these patients. Randomized controlled trials are needed to confirm this observation.

## Additional file

10.1186/s13613-016-0128-6 Additional online supplement.
