# Supplementary material for: Poor outcome is associated with less negative fluid balance in patients with aneurysmal subarachnoid hemorrhage treated with prophylactic vasopressor-induced hypertension
Source: Ann Intensive Care. 2016 Mar 31;6:25. doi: 10.1186/s13613-016-0128-6 (PMC4816937; doi:10.1186/s13613-016-0128-6)
Supplement: Supplementary file 1 — 10.1186/s13613-016-0128-6 Additional online supplement. [file 13613_2016_128_MOESM1_ESM.doc]

**Poor Outcome Is Associated with Less Negative Fluid Balance in Patients with Aneurysmal Subarachnoid Hemorrhage Treated with Prophylactic Vasopressor-Induced Hypertension**

Yasser Sakr MD PhD^1^, Pedro Dünisch MD^2^, Clesar Santos MD^3^, Lena Matthes MD^2^, Mohamed Zeidan MD, Konrad Reinhart MD^1^, Rolf Kalff MD^2^, and Christian Ewald MD^2^

**Additional File 1**

Table S1. Criteria for the Hunt and Hess and Fisher scores

| **Hunt and Hess Score** | **Criteria** |
| --- | --- |
| Grade 1 | Mild headache with no other neurological findings. |
| Grade 2 | Severe headache with neck stiffness but no neurologic deficit, except for a cranial nerve palsy. |
| Grade 3 | Impaired consciousness, drowsy or confused, with mild focal neurologic deficits. |
| Grade 4 | Stuporous, moderate or severe hemiparesis. |
| Grade 4 | Comatose patient with decerebrating posturing. |
| **Fisher scale** |  |
| Grade 1 | No sign of SAH or intraventricular hemorrhage (IVH). |
| Grade 2 | Minimal or thin SAH with no IVH. |
| Grade 3 | Thick SAH with no IVH in either lateral ventricle. |
| Grade 4 | Thick SAH and IVH in both lateral ventricles. |

Table S2. Characteristics of the aneurysms and surgical interventions

| Number of ruptured aneurysms, n (%) | 143* |
| --- | --- |
| Localization, n (%) |  |
| Anterior communicating artery | 67 (46.8) |
| Internal carotid artery | 26 (18.2) |
| Middle cerebral artery | 23 (16.1) |
| Anterior cerebral artery | 9 (6.3) |
| Others† | 18 (12.6) |
| Territory, n (%) |  |
| Anterior circulation | 125 (87.4) |
| Posterior circulation | 18 (12.6) |
| Surgical treatment‡, n (%) |  |
| Clipping | 102¶ (71.8) |
| Coiling | 32 (22.5) |
| Wrapping | 3 (2.1) |
| External ventricular drainage, n (%) | 131ǀ (92.3) |
| Craniectomy, n (%) | 45‖ (31.7) |
| VP-Shunting, n (%) | 70 (49.3) |

IQ, Interquartile range; VP-Shunt, Ventriculoperitoneal Shunt

* including one patient with two ruptured aneurysms.

† Basilar artery, posterior cerebral artery, posterior communicating artery, vertebral artery.

‡including 6 patients with clipping after 24 hours, and 4 with coiling after 24 hours.

¶ including 10 patients with incision of the lamina terminalis.

ǀ 130 patients within 24 hours, 1 patient within 48 hours.

‖ 34 patients within 24 hours, 9 patients within 7 days, 2 patients after 7 days.

Table S3. Characteristics of the study population according to the Glasgow Outcome Score (GOS)

|  | GOS after 3 months | | GOS after 6 months | | GOS after 12 months | |
| --- | --- | --- | --- | --- | --- | --- |
|  | GOS 1-3 | GOS 4-5 | GOS 1-3 | GOS 4-5 | GOS 1-3 | GOS 4-5 |
| Age, years, mean ± SD | 58 ± 14 | 48 ± 10‡ | 59 ± 14 | 48 ± 11‡ | 59 ± 13 | 48 ± 12‡ |
| Sex, female, n (%) | 55 (66.3) | 34 (57.6) | 50 (64.9) | 39 (60.0) | 48 (64.9) | 41 (60.3) |
| Hunt and Hess Score, median (IQ) | 4 (3-5) | 2 (2-3)‡ | 4 (3-5) | 2 (2-3)‡ | 4 (3-5) | 2 (2-3)‡ |
| WFNS Score, median (IQ) | 4 (2-5) | 2 (1-2)‡ | 4 (2-5) | 2 (1-3)‡ | 4 (2-5) | 2 (1-3)‡ |
| Fisher Score, median (IQ) | 4 (4-4) | 3 (2-4)‡ | 4 (4-4) | 3 (2-4)‡ | 4 (4-4) | 3 (2-4)‡ |
| Localization of aneurysms, n (%) |  |  |  |  |  |  |
| Anterior communicating artery | 35 (42.2) | 32 (54.2) | 31 (40.3) | 36 (55.4) | 29 (39.2) | 38 (55.9) |
| Internal carotid artery | 15 (18.1) | 10 (16.9) | 14 (18.2) | 11 (16.9) | 14 (18.9) | 11 (16.2) |
| Middle cerebral artery | 14 (16.9) | 9 (15.3) | 13 (16.9) | 10 (15.4) | 12 (16.2) | 11 (16.2) |
| Anterior cerebral artery | 6 (7.2) | 3 (5.1) | 6 (7.8) | 3 (4.6) | 6 (8.1) | 3 (4.4) |
| Others¶ | 13 (15.6) | 5 (8.5) | 13 (16.8) | 5 (7.7) | 13 (17.6) | 5 (7.3) |
| Treatment, n (%) – Clipping | 59 (71.1) | 43 (72.9) | 54 (70.1) | 48 (73.8) | 51 (68.9) | 51 (75.0) |
| - Coiling | 16 (19.3) | 16 (27.1) | 16 (20.8) | 16 (24.6) | 16 (21.6) | 16 (23.5) |
| - Wrapping | 2 (2.4) | 1 (1.7) | 2 (2.6) | 1 (1.5) | 2 (2.7) | 1 (1.5) |
| External ventricular drainage, n (%) | 82 (98.8) | 49 (83.1)† | 76 (98.7) | 55 (84.6)† | 73 (98.6) | 58 (85.3)† |
| Craniectomy, n (%) | 40 (48.2) | 5 (8.5)‡ | 39 (50.6) | 6 (9.2)‡ | 37 (50.0) | 8 (11.8)‡ |
| VP-Shunt implant, n (%) | 49 (59.0) | 21 (35.6)† | 45 (58.4) | 25 (38.5)* | 43 (58.1) | 27 (39.7)* |

GCS, Glasgow Coma Scale; GOS, Glasgow Outcome Score; IQ, Interquartile range; SD, Standard deviation; VP-Shunt, Ventriculoperitoneal Shunt; WFNS, World Federation of Neurological Surgeons.

* p 0.05-0.01; † p <0.01-0.001; ‡ p <0.001;

¶ Other localizations: Basilar artery, Posterior cerebral artery, Posterior communicating artery, Vertebral artery.

Table S4. Complications according to the Glasgow Outcome Score (GOS)

|  | GOS after 3 months | | GOS after 6 months | | GOS after 12 months | |
| --- | --- | --- | --- | --- | --- | --- |
|  | GOS 1-3 | GOS 4-5 | GOS 1-3 | GOS 4-5 | GOS 1-3 | GOS 4-5 |
| Hydrocephalus, n (%) | 61 (73.5) | 22 (37.3)‡ | 56 (72.7) | 27 (41.5)‡ | 54 (73.0) | 29 (42.6)‡ |
| Cerebral infarction |  |  |  |  |  |  |
| n (%) | 61 (73.5) | 20 (33.9)‡ | 58 (75.3) | 23 (35.4)‡ | 56 (75.7) | 25 (36.8)‡ |
| Territory, n (%) |  |  |  |  |  |  |
| - Anterior cerebral artery | 42 (68.9) | 8 (40.0)‡ | 42 (72.4) | 8 (34.8)‡ | 40 (71.4) | 10 (40.0)‡ |
| - Middle cerebral artery | 53 (86.9) | 14 (70.0)‡ | 50 (86.2) | 17 (73.9)‡ | 48 (85.7) | 19 (76.0)‡ |
| - Posterior cerebral artery | 10 (16.4) | 0 (0.0)† | 10 (17.2) | 0 (0.0)† | 10 (17.9) | 0 (0.0)† |
| Vasospasm |  |  |  |  |  |  |
| n (%) | 58 (69.9) | 35 (59.3) | 55 (71.4) | 38 (58.5) | 52 (70.3) | 41 (60.3) |
| Duration, day, median (IQ) | 10 (7-17) | 8 (5-14) | 10 (7-17) | 9 (5-15) | 9 (7-17) | 9 (5-16) |
| Localization, n (%) |  |  |  |  |  |  |
| - Anterior cerebral artery | 33 (56.9) | 14 (40.0)* | 31 (56.4) | 16 (42.1)* | 28 (53.8) | 19 (46.3) |
| - Middle cerebral artery | 55 (94.8) | 32 (91.4) | 52 (94.5) | 35 (92.1) | 49 (94.2) | 38 (92.7) |
| - Posterior cerebral artery | 1 (1.7) | 0 (0.0) | 1 (1.8) | 0 (0.0) | 1 (1.9) | 0 (0.0) |
| - Basilar artery | 1 (1.7) | 0 (0.0) | 1 (1.8) | 0 (0.0) | 1 (1.9) | 0 (0.0) |
| Severity, n (%) |  | ‡ |  | ‡ |  | † |
| - Mild (≥ 120 < 160 cm/s) | 5 (9.1) | 16 (47.1) | 5 (9.6) | 16 (43.2) | 5 (10.2) | 16 (40.0) |
| - Moderate (≥ 160 < 200 cm/s) | 24 (43.6) | 15 (44.1) | 23 (44.2) | 16 (43.2) | 21 (42.9) | 18 (45.0) |
| - Severe (≥ 200 cm/s) | 26 (47.3) | 3 (8.8) | 24 (46.2) | 5 (13.5) | 23 (46.9) | 6 (15.0) |

* p 0.05-0.01; † p <0.01-0.001; ‡ p <0.001

Table S5. Use of mechanical ventilation and vasopressor agents, occurrence of infection during the ICU stay and SOFA-Scores according to the GOS

|  | GOS after 3 Months | | GOS after 6 Months | | GOS after 12 Months | |
| --- | --- | --- | --- | --- | --- | --- |
|  | GOS 1-3 | GOS 4-5 | GOS 1-3 | GOS 4-5 | GOS 1-3 | GOS 4-5 |
| Mechanical ventilation | 78 (94.0) | 44 (74.6)† | 74 (96.1) | 48 (73.8)‡ | 71 (95.9) | 51 (75.0)‡ |
| Vasopressor (Norepinephrine) |  |  |  |  |  |  |
| - n (%) | 74 (89.2) | 39 (66.1)† | 70 (90.9) | 43 (66.2)‡ | 67 (90.5) | 46 (67.6)† |
| - Dosage¶ | 0.03 (0.01-0.1) | 0.03 (0.01-0.06) | 0.03 (0.01-0.1) | 0.03 (0.01-0.06) | 0.03 (0.01-0.09) | 0.03 (0.01-0.07) |
| Infection, n (%) | 61 (73.5) | 14 (23.7)‡ | 58 (75.3) | 17 (26.2)‡ | 55 (74.3) | 20 (29.4)‡ |
| Site of infection, n (%) |  | * |  | * |  | * |
| - Respiratory | 43 (70.5) | 5 (35.7) | 42 (72.4) | 6 (35.3) | 41 (74.5) | 7 (35.0) |
| - Catether-related | 6 (9.8) | 2 (14.3) | 6 (10.3) | 2 (11.8) | 5 (9.1) | 3 (15.0) |
| - Central nervous system | 2 (3.3) | 4 (28.6) | 2 (3.4) | 4 (23.5) | 2 (3.6) | 4 (20.0) |
| - Urogenital | 3 (4.9) | 2 (14.3) | 2 (3.4) | 3 (17.6) | 1 (1.8) | 4 (20.0) |
| - Others ׀ | 7 (11.5) | 1 (7.1) | 6 (10.3) | 2 (11.8) | 6 (11.0) | 2 (10.0) |
| Severe sepsis, n (%) | 11 (18.0) | 1 (7.1)* | 11 (19.0) | 1 (5.9)† | 11 (20.0) | 1 (5.0)† |
| SOFAmax, total, mean ± SD | 10.2 ± 2.4 | 6.6 ± 3.3‡ | 10.3 ± 2.0 | 6.8 ± 3.6‡ | 10.2 ± 2.0 | 7.0 ± 3.6‡ |
| SOFAmax, without GCS, mean ± SD | 6.4 ± 2.0 | 3.9 ± 2.4‡ | 6.4 ± 1.7 | 4.1 ± 2.7‡ | 6.4 ± 1.8 | 4.3 ± 2.7‡ |
| Organ failure in the ICU, n (%) |  |  |  |  |  |  |
| - respiratory | 64 (77.1) | 16 (28.1)‡ | 60 (77.9) | 20 (31.7)‡ | 58 (78.4) | 22 (33.3)‡ |
| - hematologic | 1 (1.2) | 0 (0.0) | 0 (0.0) | 1 (1.6) | 0 (0.0) | 1 (1.5) |
| - hepatic | 1 (1.2) | 0 (0.0) | 0 (0.0) | 1 (1.6) | 0 (0.0) | 1 (1.5) |
| - cardiovascular | 75 (90.4) | 44 (75.9)* | 70 (90.9) | 49 (76.6)* | 67 (90.5) | 52 (77.6)* |
| - renal | 1 (1.2) | 0 (0.0) | 0 (0.0) | 1 (1.6) | 0 (0.0) | 1 (1.5) |

* p 0.05-0.01; † p <0.01-0.001; ‡ p <0.001

¶ µg/Kg/min

׀ Other sites: abdomen, kidney, skin/surgical wounds, blood-stream and unknown focus

**Figure S1.** Box blots representing urine output in mL over the first week in the ICU in patients with poor (Glasgow Outcome Score [GOS] <3, dark boxes) vs. good (GOS>3, light boxes) outcome at 3, 6, and 12 months after the onset of SAH.

ANOVA: 3 months: p=0.008, 6 months: p=0.013, 12 months =0.004

* p<0.05 compared to patients with good outcome.

**Figure S2** Box blots representing the central venous pressure in mmHg over the first week in the ICU in patients with poor (Glasgow Outcome Score [GOS] <3, dark boxes) vs. good (GOS>3, light boxes) outcome at 3, 6, and 12 months after the onset of SAH.

ANOVA: 3 months: p=0.019, 6 months: p=0.054, 12 months =0.049

* p<0.05 compared to patients with good outcome.
